# Mold and Stain Resistance of Bamboo Treated with Pyraclostrobin Fungicide

**DOI:** 10.3390/polym14245537

**Published:** 2022-12-17

**Authors:** Jingpeng Zhang, Mingliang Jiang, Bin Zhang, Yuzhang Wu, Xingxia Ma

**Affiliations:** 1 Research Institute of Wood Industry, Chinese Academy of Forestry, Beijing 100091, China; 2 Institute of Ecological Conservation and Restoration, Chinese Academy of Forestry, Beijing 100091, China

**Keywords:** bamboo, strobilurins, pyraclostrobin, mold resistance, stain resistance

## Abstract

Bamboo is rich in starch and sugars and can be infected by mold and stain fungi, degrading its performance, shortening its service life, and reducing its utilization value. It is crucial to investigate how to protect bamboo against mold and stain fungi. The zone of inhibition test was used to evaluate the antifungal activity of azoxystrobin, kresoxim-methyl, pyraclostrobin and 3-iodo-2-propynyl-butylcarbamate (IPBC) against stain fungi (*Botryodiplodia theobromae*, *Fusarium moniliforme*, and *Alternaria alternate*) and mold fungi (*Aspergillus niger*, *Penicillium citrinum*, and *Trichoderma viride*) to develop new chemicals to protect bamboo against stain fungi and molds. The inhibitory activity of the composite pyraclostrobin and IPBC with different ratios was evaluated. Water-based formulations of the fungi were used to treat the bamboo, and the mold and stain resistance of the bamboo was investigated at different chemical retention rates. The results showed that the antifungal activity of pyraclostrobin was significantly higher than that of azoxystrobin and kresoxim-methyl. Different degrees of inhibitory activities against the stain and mold fungi were observed, and the inhibitory activity was higher against stain fungi than against molds. The three stain fungi were completely inhibited at a 7:3 ratio of pyraclostrobin to IPBC and 0.1% concentration. As the ratio increased, the inhibitory effect against mixed mold strains improved. The control efficacy of the pyraclostrobin formulations Str-1 and Str-2 at 0.1% concentration was 100% against *Alternaria alternate* and 70.8% against *Fusarium moniliforme*. The control efficacy of the composite formulations SI-1 and SI-2 at 0.1% concentration was 100% against all three stain fungi and greater than 91.8% against the mixed mold strains. This study provides new insights into the utilization of pyraclostrobin and its composite formulations as new bamboo antifungal agents.

## 1. Introduction

Bamboo is a renewable and rapidly growing lignocellulosic plant [1]. Due to its advantages of low weight, high strength, flexibility, and versatility, bamboo has become an important engineering material, such as glued laminated bamboo, bamboo scrimber, and bamboo bundle laminated veneer lumber [1,2,3]. However, bamboo has natural defects that are susceptible to various mold and stain fungi. It is easily damaged by decay fungi and other organisms due to large amounts of starch, sugar, protein, and other nutritious substances in its tissues [4,5,6]. When bamboo is exposed to moist environmental conditions during processing and storage or is used in outdoor conditions, mold and stain fungi infection substantially affects its appearance and aesthetic quality, significantly reducing its use value and limiting outdoor uses of bamboo [7,8]. The primary mold and stain fungi affecting bamboo are Ascomycetes. The genera that reproduce asexually are *Trichoderma*, *Aspergillus*, *Penicillium*, *Fusarium*, *Alternaria*, and *Globisporium* [9]. These bamboo mold and stain fungi are very difficult to control. Therefore, it is crucial to investigate how to protect bamboo against mold and stain fungi to enable the use of bamboo products indoors and outdoors.

In recent decades, bamboo has been protected against molds and stain fungi by influencing the conditions affecting the survival of fungi or killing them by using chemicals. For example, Moso bamboo was treated with aqueous solutions with different pH values to reduce the hemicellulose content, create conditions unsuitable for mold growth, and reduce the nutrients in the bamboo to inhibit mold growth [10]. Bamboo underwent a pretreatment with ultrasonic waves to remove starches and other nutrients to prevent mildew. The bamboo treated with antibacterial paraffin had no mycelium or mildew spots on its surface after 28 d [11]. However, the most simple, effective, and easy-to-implement method to prevent mildew on bamboo are chemical agents. For instance, antimicrobial nanoparticles, including titanium dioxide (TiO_2_), silica dioxide (SiO_2_), silver (Ag) and zinc oxide (ZnO) nanoparticles, and other inorganic antifungal agents, have been used to modify bamboo and improve its mold resistance to varying degrees [12,13,14,15,16]. The most commonly used and investigated mildew inhibitors are organic agents, including 3-iodo-2-propynyl-butylcarbamate (IPBC), 2-thiocyanomethylthiobenzothia- zole (TCMTB), and isothiazolinone derivatives (DCOIT, CMIT/MIT) [17,18,19]. IPBC has the best anti-mold performance and is effective against various mold fungi. The control efficacy of IPBC was 75% against *Botryodiplodia theobromae* and 100% against mixed mold strains at a concentration of 0.1%, i.e., the active ingredient retention of the treated bamboo was 0.39 g/m^2^ [20]. Unfortunately, the long-term effectiveness of IPBC is low due to its instability in high-light and high-heat conditions [21]. It is also expensive to treat bamboo and has application restrictions. Therefore, it is necessary to develop new bamboo mildew inhibitors.

**Figure 1:**
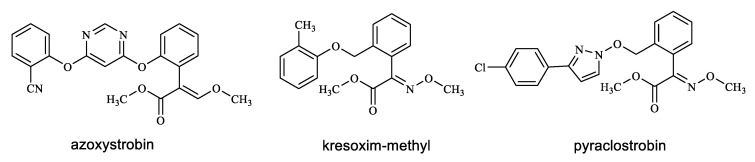
The chemical structures of strobilurin fungicides.

Strobilurin fungicides are derived from the natural antibiotic strobilurin A with fungicidal activity, which is isolated from basidiomycetes [22]. Since the discovery of its fungicidal activity, strobilurin has been structurally optimized for more than 20 years, and its biological activity has been verified, setting a new milestone in the history of fungicide development after triazole fungicides [23,24]. The mechanism of strobilurin fungicides is to inhibit the respiration of mitochondria in the cells of fungi and prevent the synthesis of energy in cells, inhibiting the germination of pathogenic fungi spores, the growth of mycelia, and the formation of spores and other growth processes [25,26]. Azoxystrobin, kresoxim-methyl, and pyraclostrobin (Figure 1) are typical representatives of strobilurin fungicides, which are characterized by high efficiency, broad spectrum, and low toxicity [23]. Furthermore, they are effective fungicides for almost all fungal infections (ascomycetes, basidiomycetes, oomycetes, and semi-fungi) [27]. A previous study investigated the inhibition of wood decay fungi (ascomycetes, basidiomycetes) using an indoor decay resistance test and demonstrated that the effectiveness of pyraclostrobin and azoxystrobin formulations was equivalent to the commercial agent propiconazole [28]. Therefore, strobilurin fungicides may be suitable for inhibiting mold and stain fungi (ascomycetes, basidiomycetes) in bamboo.

Thus, this study investigates the inhibitory activity of azoxystrobin, kresoxim-methyl, and pyraclostrobin, against bamboo stain fungi and molds using an antifungal activity test to select candidate agents and their appropriate concentrations. Several water-based agents are prepared using the candidate agents or their mixtures to treat bamboo, and the stain and mold resistance of the bamboo is evaluated. This study provides critical information for developing organic strobilurin anti-mold agents for bamboo.

## 2. Materials and Methods

### 2.1. Materials

Azoxystrobin (purity ≥ 97%), kresoxim-methyl (purity ≥ 97%), and pyraclostrobin (purity ≥ 98%) were purchased from Wenzhou Lujia Import and Export Trade Co., Ltd., Wenzhou, China. IPBC (purity ≥ 99%) was obtained from Shanghai Huilong Chemical Co., Ltd., Shanghai, China. Agricultural emulsifiers (No. 601 and No. 1601) and castor oil polyoxyethylene ether (EL-90) were purchased from Jiangsu Haian Petrochemical Co., Ltd., Nantong, China. The other reagents are commercially available and are analytical grade; they were purchased from Sinopharm Chemical Reagent Co., Ltd., Beijing, China.

A 4 years old bamboo plant (*Phyllostachys edulis*) was purchased in Hangzhou, Zhejiang Province. The bamboo was cut into specimens with dimensions of 50 mm × 20 mm × 5 mm (L × W × H) with no mildew, knots, or other defects. The moisture content of the 5 mm specimen was 8–12%. The air-dry density and moisture content of the samples were 0.71 ± 0.04 g/cm^3^ and 7.3 ± 0.2%, respectively.

The stain and mold fungi isolated from bamboo were Botryodiplodia theobromae (abbreviated to Bt), Fusarium moniliforme (Fm), Alternaria alternate (Aa), Aspergillus niger (An), Penicillium citrinum (Pc) and Trichoderma viride (Tv).

### 2.2. Methods

#### 2.2.1. Zone of Inhibition Test

The zone of inhibition test was used for screening the chemicals and their concentrations. The 6 fungal strains were cultured on a PDA substrate in Petri dishes (standard 90 mm) for 7 d at 28 °C and 85% relative humidity. We put 20 mL of distilled water into a 100 mL triangular flask and added several glass balls. The flasks were plugged with cotton plugs and autoclaved for 30 min. An inoculation needle was used to remove fragments of mycelium from the edge of the fungal colonies. They were placed into cold, sterile water in a triangular bottle, which was shaken to disperse the material and obtain the fungal suspension. Each chemical was dissolved in an aqueous ethanol solution (50%) to obtain chemical solutions with mass concentrations of 0.05%, 0.1%, and 0.2%. The fungal suspension was placed on the PDA medium, and 4 Oxford cups were placed in the Petri dishes. We used a pipette to drip 0.25 mL of the chemical solutions of various concentrations and put the Petri dishes in the incubator for culture. The test was conducted on an ultra-clean workbench; a 50% ethanol solvent served as the blank control, and the IPBC solution was a positive control. The diameter of each inhibition zone was measured by vernier caliper after 2 weeks of cultivation. Each group of tests was repeated three times, and the arithmetic mean of the test value was used.

#### 2.2.2. Selection of the Appropriate Ratio of Pyraclostrobin and IPBC

We combined 0.1 g of pyraclostrobin and 99.9 g of the 50% ethanol solution in a 200 mL conical flask under magnetic stirring for 5 min to obtain a pyraclostrobin solution with 0.1% concentration. We prepared a 0.1% IPBC solution in the same way. We used a pipette to obtain 9 mL, 8 mL, 7 mL, 6 mL, 5 mL, 4 mL, 3 mL, 2 mL, and 1 mL of the 0.1% pyraclostrobin solution and mixed it with 1 mL, 2 mL, 3 mL, 4 mL, 5 mL, 6 mL, 7 mL, 8 mL, and 9 mL of the 0.1% IPBC solution, respectively. Thus, we obtained nine mixed agents of pyraclostrobin and IPBC with a concentration of 0.1%. Their ratios were 9:1, 8:2, 7:3, 6:4, 5:5, 4:6, 3:7, 2:8, and 1:9, respectively. The zone of inhibition method was used to test the inhibitory activity of the mixed agents against *B. theobromae*, *F. moniliforme*, and *A. alternate* and the mixed mold fungi to determine the optimum ratio of pyraclostrobin and IPBC.

#### 2.2.3. Preparation of Pyraclostrobin and Composite Formulations

A mixture of 5.00 g pyraclostrobin and 20.00 g ethyl acetate was added to a 200 mL conical flask. The mixture was stirred to dissolve at 25 °C for 5 min. Then, 15.00 g of agricultural emulsifier No. 601 and 22.50 g of distilled water were dropwise added to the conical flask. The 8% pyraclostrobin formulation Str-1 was obtained after stirring. The Str-2 pyraclostrobin formulation was prepared similarly, except that the solvent was N,N-dimethylformamide, and the emulsifier was agricultural emulsifier No. 1601.

The method in reference [29] was used to prepare the 8% IPBC formulation; 2.00 g of IPBC and 9.00 g of EL-90 were added to an 8.00 g cyclohexanone solution under magnetic stirring for 10 min. Then, 6.00 g of distilled water was dropwise added to the conical flask, and the 8% IPBC formulation was obtained after stirring. Furthermore, the Str-1 formulation and IPBC formulation were mixed evenly according to the mass ratio of 7:3 and 4:6, respectively, to obtain the SI-1 and SI-2 composite formulations of pyraclostrobin/IPBC with active ingredients of 8%.

#### 2.2.4. Treatment of Bamboo with Anti-Mold Agents

According to the results of the inhibition zone test, each formulation was diluted with water to obtain mass fraction concentrations of 0.1% and 0.2%. Next, the bamboo specimens were air-dried to a moisture content of less than 12%, weighed, and marked as m1. The specimens were immersed into the chemical solutions with different concentrations and water (control) at −0.09 MPa for 30 min and were maintained at normal pressure for 30 min. The specimens were removed from the solution, the surface was wiped clean, and they were weighed and marked as m2. We calculated the amount of retained chemicals in the bamboo specimen. The specimen was air-dried to a moisture content of 8–12%. Each experiment was replicated 6 times. The amount of active ingredient retained in the bamboo was calculated by Equation (1).

*R* = (*m*_2_ − *m*_1_) *c*/*A* × 10^6^ (1)

where *R* is the active ingredient retention on the bamboo surface, g/m^2^; m_1_ is the sample weight before immersion, g; *m*_2_ is the sample weight after immersion, g; *c* is the mass fraction of active ingredients in the chemical solution; *A* is the surface area of the treated specimen, mm^2^.

#### 2.2.5. Evaluation of Mold and Stain Resistance of Treated Bamboo

The mold and stain resistance of the treated bamboo samples was evaluated according to the Chinese Standard GB/T 18261-2013 [30]. Briefly, the fungal strains were cultured on the PDA substrate in Petri dishes for 7 d at 28 °C and 85% relative humidity. After the PDA substrate was covered by the fungal strains, two sterilized glass rods (diameter of 3 mm) were placed on the PDA substrate, and two bamboo samples were placed horizontally on the glass rod. After inoculation, the fungi were cultured at 28 °C and 85% relative humidity for 4 weeks. During the incubation, the fungal growth was observed every week, and the infection area was calculated. The infection values corresponding to the infection areas are listed in Table 1. There were 6 replicate samples in each group, and the results were averaged.

The control efficacy (*E*) of the chemicals against mold infection on the bamboo samples was obtained from Equation (2).

*E* = (1 − *D*_1_/*D*_0_) × 100% (2)

where *E* is the control efficacy, %; *D*_1_ is the average infection value of the treated samples; *D*_0_ is the mean infection value of the untreated samples.

## 3. Results and Discussion

### 3.1. Preliminary Screening of Chemicals in Zone of Inhibition Test

The ability of three candidate chemicals to inhibit six molds and stain fungal strains was evaluated. Ethanol water (50%) was used as a blank control; the results showed that it did not affect the tested strains. IPBC is a highly active anti-mold agent that was used as a positive control. The results of the inhibition zone test are listed in Table 2 and Table 3.

The data suggested that the three chemicals had moderate to strong inhibitory activity against stain fungi (Table 2). Pyraclostrobin showed high inhibitory activity against three stain fungi, whereas azoxystrobin and kresoxim-methyl only had inhibitory activity against some stain fungi. Specifically, azoxystrobin had the worst inhibitory performance. It showed no inhibitory activity against *B. theobromae* and *A. alternate* at various concentrations but exhibited inhibitory activity against *F. moniliforme* at concentrations of 0.1% and 0.2%. The diameters of the inhibition zones were 10.0 mm and 22.2 mm, respectively. Additionally, there was no inhibitory activity of kresoxim-methyl against *B. theobromae*, although it showed inhibitory activity against *F. moniliforme* and *A. alternate*. The diameters of the inhibition zones were 12.1–19.8 mm and 25.0–29.6 mm, respectively. In contrast, pyraclostrobin had significant inhibitory activity against all three stain fungi, and the diameter of the inhibition zone increased with an increase in the chemical concentration. The inhibitory activity against *A. alternate* was higher than that against *B. theobromae* and *F. moniliforme*. For example, the diameters of the inhibition zones of pyraclostrobin against *B. theobromae*, *F. moniliforme*, and *A. alternate* at 0.1% concentration were 20.3 mm, 20.1 mm, and 35.9 mm, respectively. IPBC is a highly effective mildew inhibitor with outstanding inhibition activity. The diameter of the inhibition zone of IPBC against the three stain fungi was larger than 45 mm at the three concentrations.

Table 3 indicates that the three candidate agents exhibited different inhibitory activities against the molds. Pyraclostrobin showed different levels of inhibitory activities against the three molds, kresoxim-methyl inhibited some molds, and azoxystrobin had no inhibitory activity against any of the three molds. Although kresoxim-methyl did not inhibit *T. viride*, it exhibited inhibitory activity against *A. niger* and *P. citrinum* at three concentrations. The diameters of the inhibition zones were 7.8–13.5 mm and 12.4–19.6 mm, respectively. The diameter of the inhibition zone of pyraclostrobin against *P. citrinum* was 21.7–26.2 mm, and those against *A. niger* and *T. viride* were 14.3–16.0 mm and 15.7–17.9 mm, respectively, at the three concentrations. Thus, pyraclostrobin exhibited higher activity against *P. citrinum* than against *A. niger* and *T. viride*. The inhibitory activity of IPBC against the three molds was high; the inhibition zones against *A. niger* and *P. citrinum* were greater than 49 mm at 0.1% and 0.2% concentrations, significantly higher than those of the candidate agents.

In summary, pyraclostrobin exhibited higher inhibitory activity against all stain fungi and molds than the other agents. In general, its activity was slightly higher against stain fungi than against molds; thus, its ability to inhibit stain and mold fungi should be further investigated. Pyraclostrobin inhibits the respiration of mitochondria in the cells of fungi and prevents the synthesis of energy in cells, inhibiting the germination of pathogenic fungi spores [25,26]. IPBC is an organic iodine compound composed of an iodo-propyl group and butyl carbamate group. It has a strong inhibitory effect on molds, yeasts, and algae [31]. Its antifungal mechanism is related to the reaction of iodine in the molecular chain with the sulfhydryl group or hydroxyl group in the active part of the enzyme in the microbial cells, causing a loss of enzyme activity and the death of microorganisms [32]. The results of IPBC against mold and stain fungi confirmed its high efficiency, consistent with previous reports. However, IPBC is unstable under ultraviolet light and heat [21,33]. In contrast, pyraclostrobin is a stable compound; thus, the combination of pyraclostrobin and IPBC provides advantages. It is necessary to conduct further research on the combination of pyraclostrobin and IPBC.

### 3.2. Optimization of the Ratio of Pyraclostrobin and IPBC

**Figure 2:**
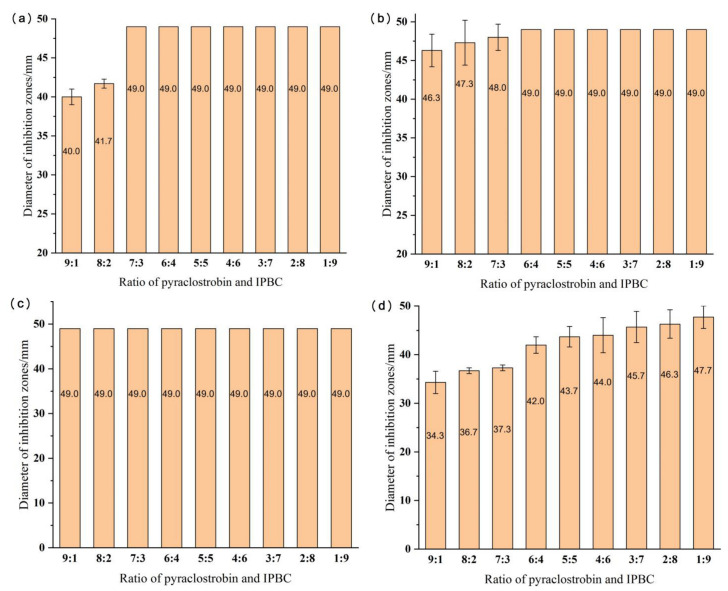
The diameter of the inhibition zone for different ratios of pyraclostrobin and IPBC at a concentration of 0.1%: (**a**) results for *Botryodiplodia theobromae*; (**b**) results for *Fusarium moniliforme*; (**c**) results for *Alternaria alternate*; and (**d**) results for mixed mold strains.

The results showed that 50% ethanol used as the blank control was inactive against the tested strains. In contrast, IPBC completely inhibited all molds and stain fungi at the concentration of 0.1% (the diameter of the inhibition zone was close to 49 mm). The concentration of the active ingredients in the chemical solution was set to 0.1%, and activity screening tests were conducted with different ratios of pyraclostrobin and IPBC to determine the optimum ratio to inhibit mold and stain fungi and minimize the amount of IPBC. The fungicidal activity against a mixture of three mold strains was tested. The results are shown in Figure 2.

The results showed that the combination of pyraclostrobin and IPBC exhibited a good inhibitory effect against the tested strains. At a ratio of 9:1, the diameters of the inhibition zones against *A. alternate* and *F. moniliforme* were more than 49 mm and 46.3 mm, respectively, higher than the diameters of the 0.2% pyraclostrobin or 0.05% IPBC solutions. The three stain fungi were completely inhibited at a ratio of 7:3, whereas the inhibitory effect on the mixed mold strains was slightly weaker, with a diameter of the inhibition zone of 37.3 mm. As the ratio increased, the inhibitory effect against the mixed mold strains increased, but they were not completely inhibited. Therefore, the concentration of the active ingredients can be increased to achieve complete inhibition of molds in bamboo. According to the results of this experiment, two ratios (7:3 and 4:6) of pyraclostrobin and IPBC were selected to treat the bamboo.

### 3.3. Stain and Mold Resistance of Bamboo Treated with Different Pyraclostrobin Formulations 

**Figure 3:**
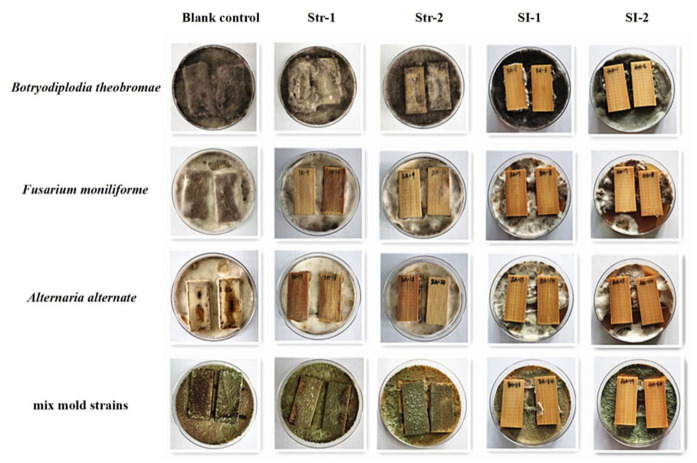
Treated bamboo specimens after mold and stain resistance tests.

The test results of the stain and mold resistance of bamboo treated with two pyraclostrobin formulations (Str-1 and Str-2) and two composite formulations with different ratios (SI-1 and SI-2) are listed in Table 4 and Table 5. The images of bamboo treated with the agents at 0.1% concentration after infection are shown in Figure 3.

The specimens in the blank control group were infected by all stain fungi, indicating no control efficacy on the three stain fungi. As illustrated in Table 4, the control efficacy of Str-1 and Str-2 was 100% against *A. alternate* and 70.8% against *F. moniliforme* at a concentration of 0.1%, i.e., the active ingredient retention of the treated bamboo was 0.36 g/m^2^. The chemical retention of the treated bamboo increased with an increase in the concentration of the active ingredient. The formulations Str-1 and Str-2 did not inhibit *B. theobromae* at a retention of 0.65 g/m^2^ (0.2% concentration). However, the surfaces of the samples treated with formulations SI-1 and SI-2 at the concentration of 0.1% (retention of 0.40 g/m^2^) had no mycelia and mildew spots (Figure 2), indicating control efficacy of 100% against the three stain fungi.

The bamboo specimens in the blank control group were infected by the mixed mold strains, indicating no control efficacy on the mixed mold strains. The data in Table 5 suggest that the Str-1 and Str-2 had negligible control efficacy on the mixed mold strains at a high retention of 0.73 g/m^2^. This result is similar to the inhibition zone activity of pyraclostrobin against the three molds. The control efficacy of SI-1 against the mixed mold strains was 91.8% at a retention of 0.40 g/m^2^ and 100% at a retention of 0.77 g/m^2^. In contrast, the control efficacy of SI-2 was 100% at a retention of only 0.42 g/m^2^.

Overall, the results of the stain and mold resistance of the treated bamboo are consistent with the inhibition zone test results. There was no difference between the stain and mold resistance of the pyraclostrobin formulations Str-1 and Str-2, which achieved 100% control efficacy against *F. moniliforme* and *A. alternate*. Composite formulations were required to achieve the effective control of *Botryodiplodia theobromae* and the mixed mold strains. The composite formulations SI-1 and SI-2 at 0.1% concentration provided the best performance in controlling *B. theobromae* and the mixed mold strains; thus, they are suitable as anti-mildew agents. The SI-1 formulation is preferable because it contains less IPBC and is less expensive. In a future study, we will conduct an outdoor mold resistance test, investigate the leaching resistance of chemicals, and adjust the chemical concentration according to the temperature and humidity conditions of the site. The long-term efficacy and other related work require further study.

## 4. Conclusions

This study explored the antifungal activity of strobilurin fungicides against bamboo stain fungi and molds using the zone of inhibition test and mold resistance tests. The following conclusions were drawn:

(1) Pyraclostrobin had higher antifungal activity than azoxystrobin and kresoxim-methyl. It exhibited different degrees of inhibitory activity against all stain fungi and molds in this test. In general, its activity was slightly higher against stain fungi than against mold fungi.
(2) The combination of pyraclostrobin and IPBC had a good synergistic effect. It completely inhibited the three stain fungi at a concentration of 0.1% and a ratio of pyraclostrobin to IPBC of 7:3. The diameter of the inhibition zone was 37.3 mm in the test with the mixed mold strains.
(3) The mold and stain resistance of bamboo treated with pyraclostrobin formulations Str-1 and Str-2 were the same, and the control efficacy increased with an increase in the concentration of the active ingredient. The composite preparations SI-1 and SI-2 had 100% control efficacy at concentrations of 0.1% against the three stain fungi and 91.8% and 100% control efficacy, respectively, against the mold strains.

## Figures and Tables

**Table 1:** Classification of the infection value.

Infection Value	Infection Area
0	No hyphae on surface
1	Infection area < 1/4
2	Infection area 1/4~1/2
3	Infection area 1/2~3/4
4	Infection area > 3/4

**Table 2:** The result of the inhibition zone tests for stain fungi.

Chemical	Mass Concentration (%)	Diameters of Inhibition Zones/mm		
		*Bt*	*Fm*	*Aa*
Azoxystrobin	0.05	0	0	0
	0.1	0	10.0 ± 2.3	0
	0.2	0	22.2 ± 2.1	0
Kresoxim-methyl	0.05	0	12.1 ± 3.0	25.0 ± 4.9
	0.1	0	13.1 ± 1.3	27.1 ± 4.7
	0.2	0	19.8 ± 3.6	29.6 ± 4.1
Pyraclostrobin	0.05	16.0 ± 2.7	17.7 ± 3.1	28.6 ± 1.5
	0.1	20.3 ± 3.2	20.1 ± 1.1	35.9 ± 2.5
	0.2	24.6 ± 2.2	24.6 ± 3.4	39.3 ± 1.3
Iodopropynyl butyl carbamate (IPBC)	0.05	44.7 ± 2.6	45.8 ± 2.4	45.4 ± 1.5
	0.1	46.9 ± 2.6	>49	>49
	0.2	>49	>49	>49

**Table 3:** The result of the inhibition zone tests for mold fungi.

Chemical	Mass Concentration (%)	Diameters of Inhibition Zones/mm		
		*An*	*Pc*	*Tv*
Azoxystrobin	0.05	0	0	0
	0.1	0	0	0
	0.2	0	0	0
Kresoxim-methyl	0.05	7.8 ± 0.4	0	12.4 ± 0.5
	0.1	10.9 ± 0.5	0	14.7 ± 0.7
	0.2	13.5 ± 0.8	0	19.6 ± 0.8
Pyraclostrobin	0.05	14.3 ± 0.7	15.7 ± 0.4	21.7 ± 1.9
	0.1	15.9 ± 0.5	16.6 ± 0.5	22.6 ± 1.6
	0.2	16.0 ± 0.7	17.9 ± 0.4	26.2 ± 3.4
IPBC	0.05	37.6 ± 1.6	35.9 ± 0.4	44.9 ± 3.6
	0.1	>49	39.1 ± 1.0	>49
	0.2	>49	41.4 ± 1.9	>49

**Table 4:** Stain resistance of bamboo treated with selected chemicals.

Chemical	Concentration (%)	*Bt*			*Fm*			*Aa*		
		*R*/(g·m^−2^)	*D*	*E* (%)	*R*/(g·m^−2^)	*D*	*E* (%)	*R*/(g·m^−2^)	*D*	*E* (%)
Str-1	0.1	0.33	4	0	0.32	0.67	83.3	0.40	0	100
	0.2	0.65	4	0	0.63	0.33	91.8	0.80	0	100
Str-2	0.1	0.35	4	0	0.36	1.17	70.8	0.36	0	100
	0.2	0.69	4	0	0.71	0.5	87.5	0.70	0	100
SI-1	0.1	0.41	0	100	0.40	0	100	0.39	0	100
	0.2	0.76	0	100	0.79	0	100	0.85	0	100
SI-2	0.1	0.39	0	100	0.38	0	100	0.40	0	100
	0.2	0.67	0	100	0.77	0	100	0.80	0	100

Note: *R*: Chemical retention; *D*: Infection value; *E*: Control efficacy.

**Table 5:** Mold resistance of bamboo treated with selected chemicals.

Chemical	Concentration (%)	Mm (Mixed Mold Strains)		
		*R*/(g·m^−2^)	*D*	*E* (%)
Str-1	0.1	0.39	4	0.0
	0.2	0.73	4	0.0
Str-2	0.1	0.36	3.83	4.3
	0.2	0.73	3.5	12.5
SI-1	0.1	0.40	0.33	91.8
	0.2	0.77	0	100
SI-2	0.1	0.42	0	100
	0.2	0.79	0	100

Note: *R*: Chemical retention; *D*: Infection value; *E*: Control efficacy.

## Data Availability

The date presented in this study are available on request from the corresponding author.
